# Treatment analysis environment for review of MRgHIFU treatments: a multi-parametric analysis tool

**DOI:** 10.1186/2050-5736-3-S1-O102

**Published:** 2015-06-30

**Authors:** Marijn van Stralen, Roel Deckers, Wilbert Bartels, Clemens Bos, Chrit Moonen

**Affiliations:** 1 University Medical Center Utrecht, Utrecht, Netherlands

## Background/introduction

Magnetic resonance imaging (MRI) provides a wide spectrum of imaging contrasts, which have utility for preparation and evaluation of HIFU treatments, as well as for guiding HIFU energy delivery (MRgHIFU). Currently, research into the prospective value of these imaging markers and their ability to predict and probe treatment success is challenged by the lack of an analysis environment that is able to process the spectrum of imaging and treatment data as a whole, and allows assessing correlation between data sets. As an example, we may want to relate the placement and temperature evolution of sonication cells with the resulting non-perfused volume, assessed by MRI perfusion imaging.[1] An important factor in this issue is that treatment data is poorly accessible to researchers. E.g. for the Philips Sonalleve platform, treatment data is almost exclusively available in log files and thermometry images are exported in a proprietary format (Par/Rec). We propose an analysis environment for clinical and basic imaging research in the context of MRgHIFU treatments.

It will be made available to the research community to i) accelerate developments in the field, ii) enable effective use of advanced imaging in MRgHIFU research and iii) stimulate collaboration between centers.

## Methods

We set up a modular framework for the analysis of MRgHIFU treatments. Tools to access treatment and imaging data were implemented, including DICOM image data and segmentations, treatment data such as treatment cell geometry and parameters, and importantly, intra-operative thermometry data. Next, image registration of these data was implemented and analysis tools were developed that enable correlation of treatment data with imaging parameters based on the targeted treatment geometries. These analysis tools were built, based on the MeVisLab medical imaging development environment (MeVis Medical Solutions, Bremen, Germany), allowing easy distribution among research sites. We demonstrate its functionality and value in clinical and basic science in the analysis of uterine fibroid treatments.

## Results and conclusions

**Figure 1:**
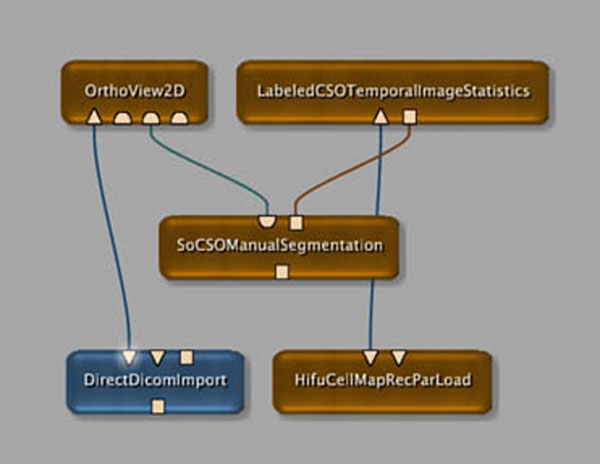
A screenshot of the modular development environment that is easily extendible with new modules.

**Figure 2:**
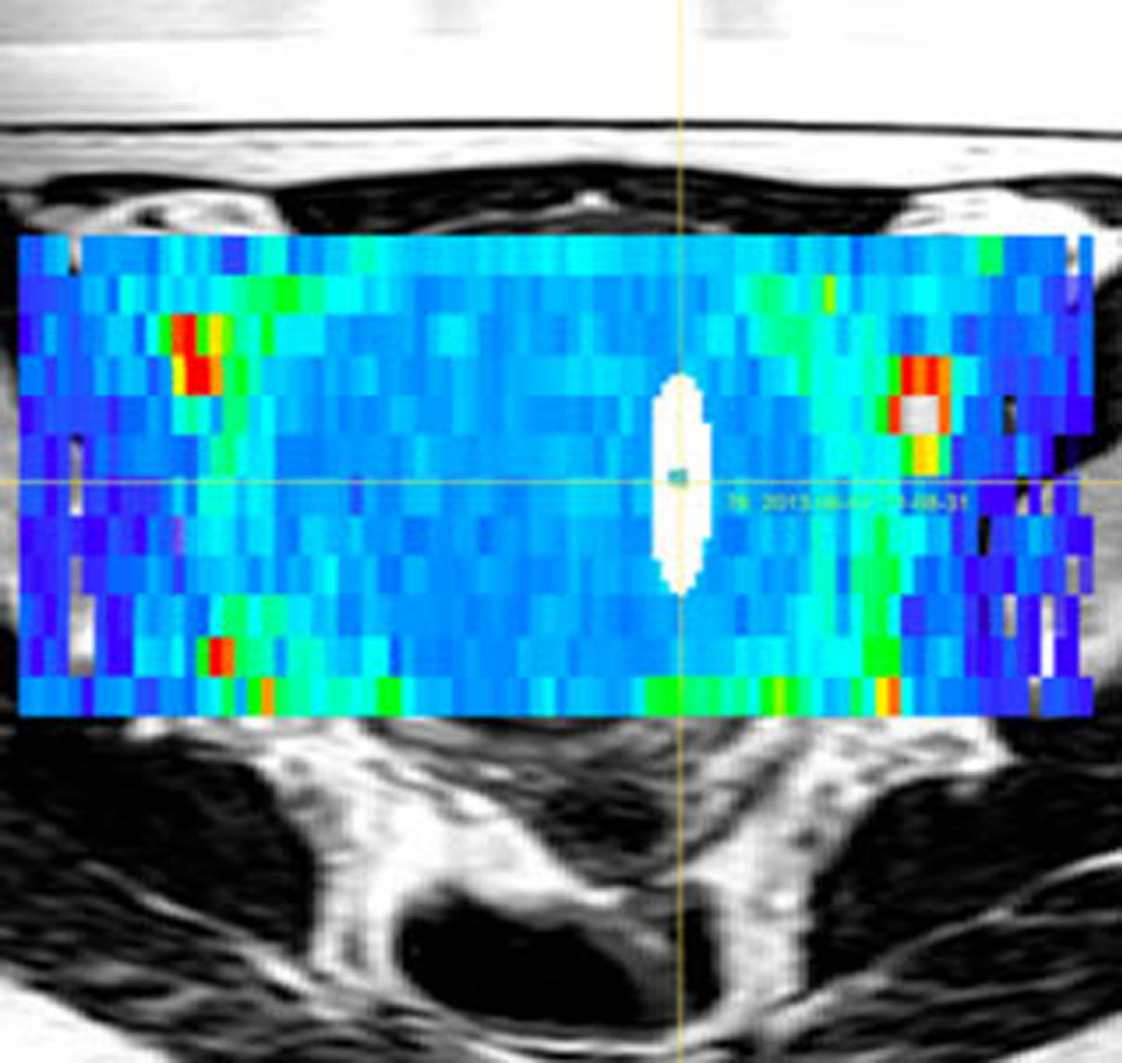
Visualization and regional image analysis for multi-parametric treatment analysis: relating treatment cell geometry (white) to an apparent diffusion coefficient (ADC) map (colored), overlaid on a planning T2w scan.

**Figure 3:**
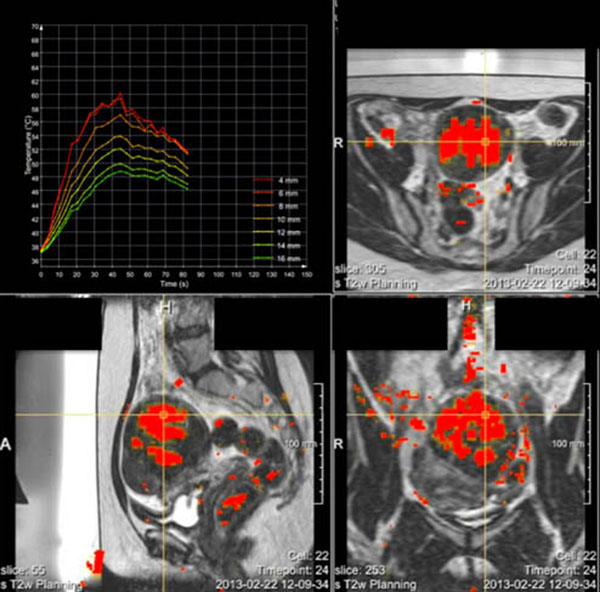
Temporal analysis of thermometry data. The three orthogonal views show cumulative thermal dose maps. The upper left graph displays temperature curves within a treatment cell, for coaxial ellipsoids with varying diameter.

After import of the relevant pre-, per- and post treatment HIFU and imaging data, the analysis environment successfully registered the data for combined analysis. Fig. 1 shows a screenshot of the modular framework of the analysis tools, which easily allows extension to new imaging methodology or treatment possibilities. Fig. 2 demonstrates the ability to correlate treatment cells to various image-based tissue parameter maps, such as diffusion parameters. The temporal analysis of the thermometry images is shown in Fig. 3. The provided toolset extends functionality that is available on the treatment console and allows further extensions by its modular framework. Also interfaces to popular existing data processing packages, such as Matlab, ITK, VTK and python are included. We intend to make the tools available for research use at the time of the symposium aiming at further development of the analysis tools as a community effort. This could accelerate research into the role of imaging in patient selection, treatment planning, as well as prediction and evaluation of treatment success, as was for example assessed for uterine fibroids.[2]

## References

[B1] Voogt Volumetric feedback ablation of uterine fibroids using magnetic resonance-guided high intensity focused ultrasound therapy European Radiology 2012 22 2 411 417 10.1007/s00330-011-2262-8 21901565 PMC3249029

[B2] Funaki Subjective effect of magnetic resonance-guided focused ultrasound surgery for uterine fibroids J Obstet Gynaecol Res 2007 33 6 834 9 10.1111/j.1447-0756.2007.00665.x 18001451

